# Texas Fever and Its Relation to the Live-Stock Interests of Tennessee1Read before the Thirty-ninth Annual Meeting of the American Veterinary Medical Association, held in Minneapolis, Minn., September 2, 3, 4, and 5, 1902.

**Published:** 1902-10

**Authors:** W. C. Rayen

**Affiliations:** Nashville, Tenn.


### TEXAS FEVER AND ITS RELATION TO THE LIVE-STOCK INTERESTS OF TENNESSEE.[1]

By W. C. Rayen, D.V.S.,
NASHVILLE, TENN.

In a discussion of the subject of Texas fever and its relation to the live-stock interests of Tennessee I shall not attempt to refer to the scientific features of the disease, for every veterinarian is now fully informed in regard to the progress that has been made up to the present time. I shall, however, confine my efforts to a discussion of the conditions that have prevailed in Tennessee for a number of years and the methods that have been pursued up to this time by the State authorities in the management of communicable diseases among domestic animals, and Texas fever in particular, with the hope that I may be able to secure from this intelligent convention of veterinarians some ideas and suggestions that will eventually contribute toward a more rational and practical application of the information we now possess in regard to this disease. The position occupied by Tennessee in relation to Texas fever is somewhat different from that of many other Southern States, from the fact that the State is exposed to and adjoins a much larger portion of the permanently infected area, and that

[1] Read before the Thirty-ninth Annual Meeting of the American Veterinary Medical Association, held in Minneapolis, Minn., September 2, 3, 4, and 5, 1902.

only a certain area in the State is more or less infected. A quarantine line described and supposed to be maintained by the State divides what is known as the infected area from the counties that are said to be free from infection. This quarantine line commences at the northwest corner of Shelby County on the Mississippi River, and extends east in a zigzag manner throughout the State from west to east to the northeast corner of Polk County, and in its course affects thirty counties in the southern and central portions of the State.

The topographical conditions of Tennessee are, perhaps, as varied as can be found in any other given area east of the Mississippi River. Commencing at the North Carolina line at an elevation of some five thousand feet above the level of the sea, there is a gradual fall from east to west in a distance of about five hundred miles to the Mississippi River, where the elevation is only two hundred feet.

In any section where such radical changes in elevation take place there must necessarily be a vast diversity in the fertility and adaptability of the soil for agricultural purposes. Such are certainly the conditions found in Tennessee. Large areas of land, both mountain and otherwise, are, owing to the quality and condition of the soil, but sparsely settled, without any enclosures to speak of, and utilized practically for pasturage purposes alone. These vast areas in Tennessee are in most instances covered with what is known in the South as sorry or stunted timber, and during the spring, summer, and fall with nutritious grasses of various varieties that afford abundant pasturage for cattle and sheep, and they are principally devoted to that purpose. In Tennessee these lands are called flat woods or barrens in the lower sections, while on the mountains in the eastern portion of the State they are known as "the range."

It is this open land throughout the State that is more or less infested with Boophilus bovis, and from this fact is considered permanently infected with Texas fever. As these lands approach sections where the country is more thickly populated, the lands enclosed by fences, the ticks disappear and the country seems to be as free from their presence as similar territory in the North. Many of the counties in what is known as the infected area are composed of large numbers of enclosed farms that in some instances cover a very large portion of the county, that are entirely free from infection, and cattle raised on such farms are always susceptible to Texas fever.

In a State so abundantly supplied with lands that can only be utilized at the present time for pasturage purposes it can be readily seen that the subject of Texas fever among cattle is of paramount importance to the agricultural population, and is a problem that requires intelligent and earnest attention if the conditions that prevail are to be removed. While the efforts that have been made by the Federal authorities have been of great benefit to the States north of the quarantine line, and to the cattle industry of the entire country, they have, in a State located on the border as Tennessee is, made the profitable breeding and raising of high-class beef-producing cattle almost an impossibility in many sections of the State. I am informed that one county that is composed of a very large area of range land in the mountain regions, which now sends to the market an average of about twelve hundred cattle annually, could in a short time, if there were no restrictions placed on the traffic, easily produce five thousand marketable cattle each year; and many other counties in what is known as the permanently infected area of the State are in a similar condition. In a State where the natural conditions are so favorable for the economical production of live-stock; where the fertile lands at the foot of the mountains and the valleys that follow the streams produce an abundance of grain and dry forage for feeding animals during the short mild winters; where the mountain ranges with their luxuriant grasses will furnish an abundance of nourishment for nearly nine months in a year for thousands of animals, this condition should not be allowed to continue. It is not this section alone that suffers, for in every part of the State there is a feeling of fear of this disease that prevents the agricultural population from engaging in the production of beef-producing animals.

It is simply impossible under existing conditions to estimate the effect produced on the cattle industry of Tennessee by the infesting agents of this disease. Persons who are engaged in the breeding and raising of live-stock usually have trouble enough in other directions to overcome, but when we add the losses that annually occur from Texas fever among susceptible cattle, and reduction in prices they are obliged to accept on account of their cattle coming from a section that is more or less infected, the importance of how to improve the present condition becomes food for serious thought.

The task of educating the agricultural population of the State on a subject of such vital importance to their interests would seem to be the first effort that should be attempted, and how to accomplish this is the problem that must be solved if better conditions are to be expected.

In Tennessee only feeble efforts have been made as yet in the direction of cultivating a better understanding of this disease and its management. For a number of years the United States Department of Agriculture have had trained inspectors stationed in Tennessee, who have given what assistance they could in the management of the quarantine line, and at the same time they have made consistent efforts to bring about a better understanding of the conditions that exist. With the limited authority that is given them within the boundary lines of the State they have certainly done everything they can, but progress under the conditions that prevail and confront them is extremely slow. The first legislation covering the entire field of communicable diseases among domestic animals in Tennessee was enacted in 1893, and the management and control were placed with the State Board of Health through a State veterinarian, but the Legislature failed to provide an appropriation for the work and very little was accomplished. Not until 1897 were the State authorities provided with an appropriation for the work; from that period the State Board of Health made some feeble efforts through inspectors who had no training, and practically disposed of the State veterinarian by placing him upon a salary on which he could not be expected to be of any service. This condition of affairs continued until 1901, when the management of communicable diseases among domestic animals was transferred by the Legislature to the Bureau of Agriculture, and the provisions of the transfer provided for the appointment of a State live-stock inspector and three assistant inspectors; there was also a provision that the chief inspector might employ a veterinarian when he thought necessary. The appropriation for the inspectors was four thousand dollars per annum, while three hundred dollars was placed at their disposal for the purpose of employing a veterinarian. Under the provisions of this legislation a farmer and stock-dealer was appointed to take charge of communicable diseases among domestic animals, and he in turn appointed three assistant inspectors who were all without experience or knowledge of such work

The further provisions of the laws of Tennessee grant every facility and authority for the organization of a thorough and systematic sanitary service, and if intelligently directed could be expected to accomplish positive results; almost unlimited authority is given the Commissioner of Agriculture and the State live-stock inspector as the directing head of such an organization, while the county board of health of each county is made a local arm for the purpose of carrying out any instructions or rules and regulations they may issue on any subject that may arise, and the cost of enforcing such rules and regulations must be paid by each county in which their application becomes necessary.

Like all other sanitary subjects, if results are to be expected success must depend upon an intimate knowledge of the diseases of animals, and requires the attention and direction of persons of scientific training and education, and this the laws of Tennessee have never adequately provided for. For a number of years veterinarians of the State have endeavored to impress upon those in charge of the work the relation that should exist between the live-stock industry and the veterinarian, but little progress has been made. If the information obtained through scientific investigation is ever utilized for the benefit of the live-stock industry in the area now regarded as permanently infected with Texas fever, with a view of accomplishing satisfactory results, there must be some radical changes in the management of communicable diseases among domestic animals. With but few exceptions, the States in the South are in much the same condition as Tennessee in regard to the prevention of animal diseases, and some are even without any legislation. There should be uniform legislation on the subject by the States now affected with Texas fever that would provide for thorough organization, intelligent direction, co-operation with each other and the Federal authorities.

If this could be accomplished, then with the assistance that would be given by the Federal Government, some progress might be made in the eradication of this disease, and thereby place the live-stock industry of the South on an equal footing with the balance of the country. If it would be found that in some sections the destruction of the tick would be impossible or not practical, in such cases local restrictions and regulations could be put in force that would permit the shipment of cattle to market without the least danger to the cattle industry north of the quarantine line, and such procedure would stimulate a desire on the part of the agricultural population of the South to co-operate in the effort to remove the infesting agents of this disease. There is a large part of the infected area in Tennessee from which, with proper supervision, cattle could now be shipped to the North during any season of the year without the least danger of communicating Texas fever.

But let me say in conclusion, that there is back of this, as there is in many vital questions, this proposition, whether the powers that be in our government, national, State, municipal, and county, shall or shall not recognize scientific training; whether we cannot by the strength of our reason and the intelligence and energy of our acts shame the people of every State in which overpaid ignorance interferes with the development of an important industry, to turn out of the darkness, and bring to this higher development scientific training. In this question, as in many others of government, morals, and, in fact, almost every question which comes to us for solution, we find that the price of liberty is eternal vigilance. And so I ask, and in speaking I voice the aspirations and the wishes of a large number of the people of Tennessee, that this Association and its members do all in their power to encourage us to keep our vigils, so that in time, from this long and constant watch, unrecompensed though it be, better things will grow and better times will come, and, this accomplished, we shall be fully compensated and paid for all our services in the consciousness of duty well done. No higher reward do we desire.

